# First report of environmental isolation of *Cryptococcus* and *Cryptococcus*-like yeasts from Boyacá, Colombia

**DOI:** 10.1038/s41598-023-41994-6

**Published:** 2023-09-21

**Authors:** Briggith-Nathalia Serna-Espinosa, Maribel Forero-Castro, María Eugenia Morales-Puentes, Claudia Marcela Parra-Giraldo, Patricia Escandón, Zilpa Adriana Sánchez-Quitian

**Affiliations:** 1 Facultad de Ciencias, Universidad Pedagógica y Tecnológica de Colombia, Avenida Central del Norte 39-115, Tunja, Boyacá, Colombia; 2 Unidad de Proteómica y Micosis Humanas, Grupo de Enfermedades Infecciosas, Departamento de Microbiología, Facultad de Ciencias, Pontificia Universidad Javeriana, Bogotá, 110231 Colombia; 3 Grupo de Microbiología, Instituto Nacional de Salud, Calle 26 # 51-20, Bogotá, D.C. Colombia; 4 Grupo de Investigación Gestión Ambiental, Facultad de Ciencias e Ingeniería, Departamento de Biología y Microbiología, Universidad de Boyacá, Carrera 2ª Este No. 64–169, Tunja, Boyacá, Colombia

**Keywords:** Microbiology, Molecular biology

## Abstract

The *Cryptococcus* genus comprises more than 100 species, of which *C. neoformans* and *C. gattii* are the leading cause of cryptococcosis. The distribution of *C. gattii* and *C. neoformans* species complexes has been extensively studied and widely reported globally. Other species such as *Naganishia albida*, *Papiliotrema laurentii*, and *Papiliotrema flavescens* have been reported as pathogenic yeasts. Since there are no reports of environmental isolation in the Boyacá region (Colombia), this study aimed to isolate and characterize *Cryptococcus* and *Cryptococcus*-like yeasts from pigeon feces, *Eucalyptus*, and olive trees distributed in the municipalities of Tunja and Ricaute Alto. The environmental data was recovered, and the isolations obtained were identified by microscopy, biochemical test, MALDI-TOF MS, *URA5*-RFLP, and sequencing of the ITS and LSU loci. For the 93 pigeon dropping samples collected in Tunja, 23 yielded to *C. neoformans*, 3 to *N. globosa*, 2 *N. albida* and 1 to *P. laurentii*. Of the 1188 samples collected from olive trees, 17 (1.43%) positive samples were identified as *C. gattii* species complex (4), *C. neoformans* species complex (2), *P. laurentii* (3), *N. albida* (2), *N. globosa* (5) and *P. flavescens* (1). Likewise, specimens of *C. neoformans* presented molecular type VNI and molecular type VNII; for *C. gattii* the molecular types found were VGIII and one VGIV by *URA5*-RFLP but VGIII by MALDI-TOF and sequencing of the ITS and LSU. Therefore, it can be concluded that the species of *Cryptococcus, Naganishia and Papiliotrema* genera, are present in the environment of Boyacá, and show a predilection for climate conditions that are typical of this region.

## Introduction

The most important pathogenic species of the *Cryptococcus* genus include the *Cryptococcus neoformans* species complex and the *Cryptococcus gattii* species complexes, responsible for skin, lung, and, more frequently, central nervous system infections^1–3^. As is the case of cryptococcosis, which worldwide estimates one million positive cases and more than 181.100 deaths from it^4^, the incidence in Colombia was represented in the last study carried out in 2018 whit an annual incidence of 0.24 × 10^4^ inhabitants and in AIDS patients being 1.1 × 10^3^ inhabitants, considering *C. neoformans* VNI molecular pattern as the most prevalent (*n* = 321, 96.1%) and for *C. gattii* species complex, the most prevalent species was *C. deuterogattii* (VGII 54.3%), followed by *C. bacillisporus* (VGIII 32.6%)^5^ For the department of Boyacá this disease is mostly caused by *C.gattii* species complex, with an incidence of 3.3%^6^. The organization of these species concerning to their nomenclature has been established in two hypothesis; the classical that included two species and nine main molecular types, VNI, VNB and VNII (representing *C. neoformans* var. grubii, serotype A), VNIV (*C. neoformans*, serotype D) and VNIII (the hybrid of these two species, serotype AD), as well as VGI, VGII, VGIII and VGIV (*C. gattii*, serotype B or C), and VGII has been subclassified into three associated genotypes: VGIIa, VGIIb, and VGIIc)^7^. However, differences in genetic and population structure of this yeast are not represented by only two species, and the use the term “species complex”, proposed by Kwon-Chung et al.^8^, is crucial to represent the diversity of the etiologic agents of Cryptococcosis^8^. In this regard, the other hypothesis, strongly supported by the phylogenetic studies by Liu et al.^9^ and Hagen et al.^10, 11^ suggest the *C. neoformans* variety grubii serotype A with VNB, VNI and VNII genotypes, *C. deneoformans* (referred to as *C. neoformans* var. neoformans with serotype D y genotype VNIV) and a hybrid composed of *C. neoformans* and *C. deneoformans* (with serotype AD and genotype VNIII). For the complex of species of *C. gattii* reorganized as five species: *C. gattii* with genotype VGI, *C. deuterogattii* with genotype VGII, *C. bacillisporus* with genotype VGIII, *C. tetragattii* with genotype VGIV, and finally *C. decagattii* with genotype VGIV and the atypical molecular type VGIIIc, initially classified as *C. bacillisporus* VGIII but after being compared to ISHAM consensus Multilocus sequence typing (MLST) was clustered together with the reference strain *C. decagattii*. In addition to being hybrids such as *C. deneoformans* with *C. gattii*, the hybrid *C. neoformans* with *C. gattii* and the hybrid C. neoformans with *C. deuterogattii*^9–12^.

These species present differences in geographic distribution and habitat and have been reported in diverse environments, reflecting the adaptability of these fungi in different parts of the world^13^. It should be noted that other *Cryptococcus*-like yeasts, have been reported in human conditions, such as *Naganishia liquefaciens* (formerly *Cryptococcus liquefaciens*), which was reported to be the cause of a fungemia in Japan^14^, *Papiliotrema laurentii* (formerly *Cryptococcus laurentii*) related to a case of meningitis^15^*, Naganishia albida* (formerly *Cryptococcus albidus*) reported in a case of fungemia in an immunocompromised child^16^ and *Papiliotrema flavescens* (formerly *Cryptococcus flavescens*) that has been reported in the cerebrospinal fluid of an AIDS patient^17^. The term “*Cryptococcus*-like yeasts” denotes a cluster of yeast species that display resemblances in terms of morphological traits with the *Cryptococcus* genus but these yeast species may not be exclusive to the *Cryptococcus* genus.

The distribution of the species *C. gattii* and *C. neoformans* species complexes has been extensively studied since these generally survive in tropical and subtropical climates. The species *C. neoformans* has been associated with eucalyptus detritus^18^ almond tree bark (*Terminalia catappa*)^19^ and other species such as Olive trees (*Olea europea*)^20^, it can survive and develop thanks to the decomposing organic matter that provides it with the basic nutritional requirements^18–21^. Likewise, it is associated with avian excreta, especially pigeons (*C. livia*); this happens due to the high nitrogen, creatinine, and salts that generate a favorable environment for fungus development. It has been found that feces with low moisture content and low exposure to sunlight are a good reservoir of this species complex^22–25^. This species has been recorded in places around the world, such as Malawi^26^, Nigeria^27^, China^28^, South Africa^29^, Brazil^30^, United States^31^, Italy^32^, Argentina^33, 34^ and Ecuador^35^, among others^4, 8, 36–39^.

In addition, the main environmental sources of *C. gattii* species complex is associated to decaying wood from eucalyptus, almond, oak, rubber, olive trees, among others^20, 40–42^, in the same way, it has been found in other environments such as soil, air and water^43–45^.

In the same way different reports associate the presence of *C. gattii* species complex with regions with temperate climates and periods with higher humidity; however, it is vital to consider the specific climatic conditions of the area since the development of the fungus depends on this^46–49^. *C. gattii* s.l. has been isolated in countries such as Australia^50^, Africa^29^, India^51, 52^, Italy^53^, United States^54^ Southern California^55^, Canada^56^, Spain^9^, China^28^, among other studies^39, 40, 47, 52, 57–61^.

Environmental isolations of the other *Cryptococcus*-like yeasts species such as *N. liquefaciens* (formerly *C. liquefacien*), *P. flavescens* (formerly *C. flavescens*), have been reported in Brazil^62^, *N. albida* (formerly *C. saitoi*) was isolated from the Antarctic soil, indicating that it prevails in cold areas^63, 64^ and the species of *P. laurentii* (formerly *C. laurentii*), *Cystofilobasidiales macerans* (formerly *C. macerans*) and *N. albida* (formerly *C. albidus*) have been reported in Bogotá city, Colombia^65, 66^.

Colombia, having a spatial location influenced by the variation of bimodal climatic conditions typical of the tropics, has become, like other countries in the region, a potential area for the spread of these fungi, where species of the *C. neoformans* complex have been reported in the departments of Cauca, Córdoba, Cundinamarca, Huila, Nariño, Norte de Santander and Valle del Cauca. For the *C. gattii* complex species, the main reports are in the departments of Norte de Santander and Cundinamarca^67^.

It should be noted that the department of Boyacá has a high variability of climatic conditions, with sectors where there are 500–1000 mm of average annual rainfall, especially the region of Ricaurte Alto. This bimodal behavior is presented in the west of the department, semi-humid and temperate climates predominate in these sectors, which would positively influence the existence of the fungus in the area^49, 68^. For this reason, it is of the outmost importance to generate studies for the identification and isolation of *Cryptococcus* species in this region and encourage further research related to the presence of the fungus and its consequent transmission to human populations.

Various investigations have established that there is a relationship between human infection and exposure to environments where there is the presence of yeast^25^; therefore, it is essential to understand their distribution in the environment and generate significant contributions that help to deduce the behavior and dynamics of these species that remain in the environment, under specific climatic conditions. Therefore, the objective of this research was to establish the first report of species of the *Cryptococcus and Cryptococcus-like yeasts* in the department of Boyacá, thus contributing with new knowledge about the environmental distribution of this microorganism that generates relevant data for human health care.

## Results

### Sample collection

**Figure 1:**
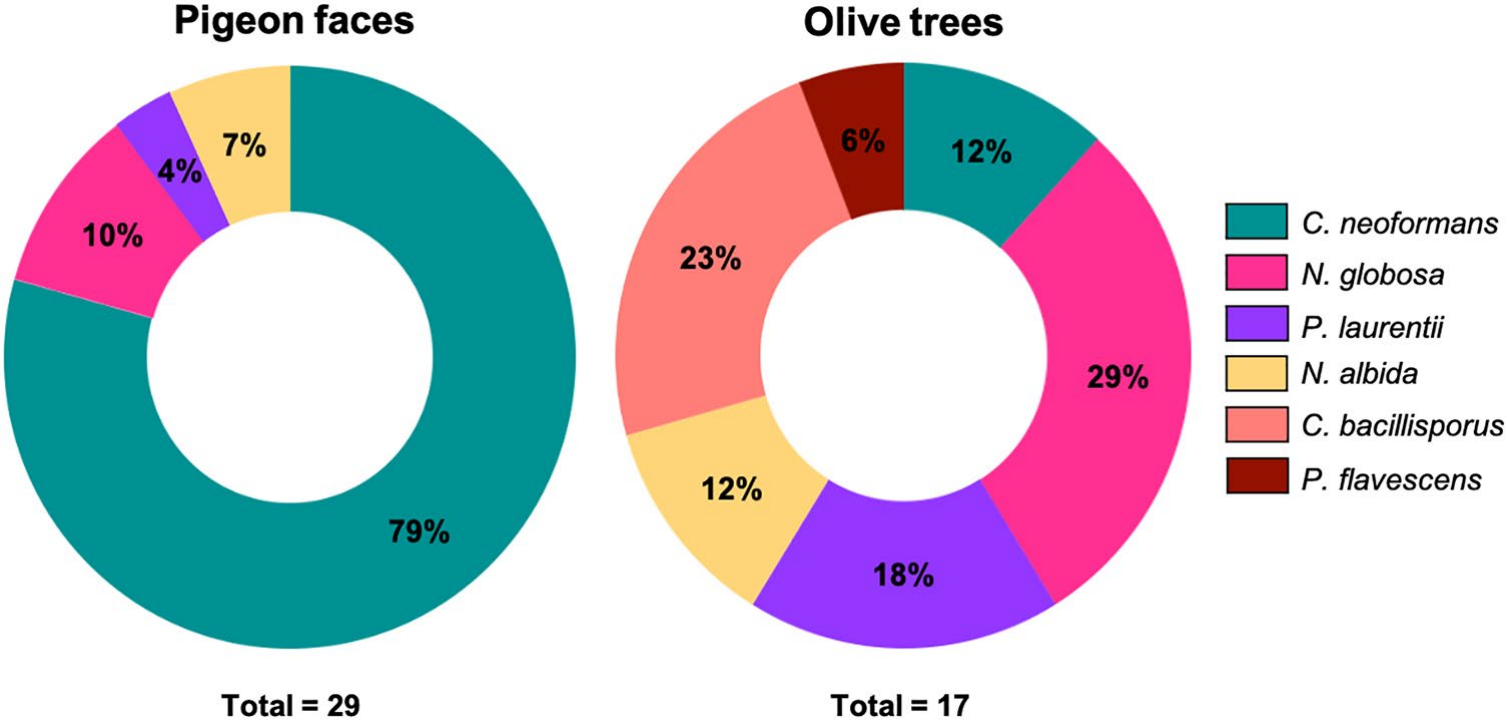
Yeasts obtained from pigeon droppings (Tunja) and olive trees (Sáchica, Sutamarchán and Villa de Leyva). (Identified by biochemical tests and molecular techniques).

Of the 93 samples recollected from pigeon feces in Tunja, 64 (68.8%) were negative, 23 (24.7%) were positive for *C. neoformans* and 6 (6.5%) for other *Cryptococcus*-like yeasts specie; 3 (3,22%) *N. globosa*, 2 (2.2%) *N. albida* and 1 (1.07%) *P. laurentii*, in addition to finding nine species of yeast from other genera. No isolates associated with *Cryptococcus* species were obtained from the 1211 eucalyptus tree samples taken in Tunja. For the 1188 environmental samples from olive trees collected, 1171 (98.57%) were negative, 4 (0.33%) were positive for *C. bacillisporus*, 2 (0.17%) for *C. neoformans*, and 11 (0,93%) for other non-neoformans cryptococcal specie such as *P. laurentii* (3), *N. albida* (2), *N. globosa* (5) and *P. flavescens* (1), in addition to finding four yeast species of other genera and two bacterial species. The distribution of the species found is presented in Fig. 1.

### Climatic and environmental characteristics of the study areas

For the feces sampling of *C. livia*, variables of solar brightness, relative humidity, evaporation, precipitation, and temperature were taken; where a maximum temperature of 13.8 °C and a minimum of 12.1 °C was evidenced, maximum solar brightness recorded was 180 h per month, relative humidity of 81.8%, observing the highest rainfall in April with 174.7 mm (Supplementary Table 1). The data obtained from the sampling points included variables of maximum, minimum, and average temperature, precipitation, relative humidity, species collected, UV radiation variables, such as direct and indirect sunlight, photosynthetic photon flux density (PPFD), direct and indirect, detailed information in the global data matrix (supplementary table 2).

Within the variables recorded in the sampling associated with olive trees, the maximum temperature recorded was 25 °C and a minimum temperature of 9 °C. In addition, an increase in relative humidity (maximum of 75.8%) was recorded in the municipality of Sutamarchán During July. The most recurrent direct light indices were low, with a value of 0.0394 and indirect light 0.0173.

### Microbiological identification

**Figure 2:**
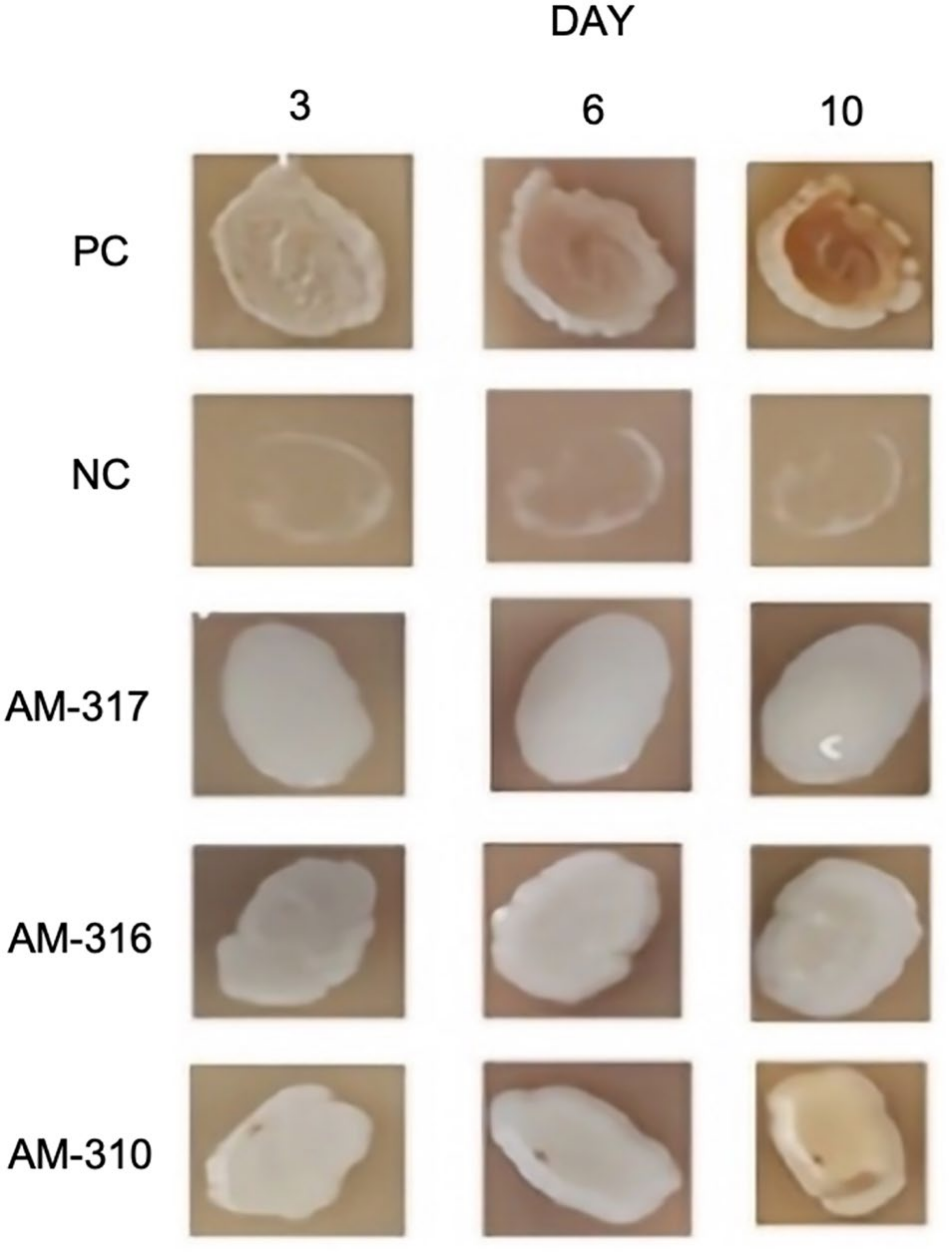
Phenoloxidase activity of *C. bacillisporus* (AM-316, AM-317) and *C. neoformans* (AM-310). Negative control (NC) *C. albicans*, and positive control (PC) *C. neoformans* ATCC 32,045.

The results of the microbiological identification showed that 45 of 46 presented a capsule. In addition, 35 of the 46 isolates were positive for the urease test, and 42 of the 46 isolates grew at 37ºC, except for three species of *N. albida* (AM-0277, AM-0286, and AM-0323), and one of *N. globosa* (AM-0329). All isolates corresponding to *C. bacillisporus* (AM-0310, AM-0316, AM-0317 and AM-0333) were positive in CGB (L-canavanine-glycine-bromothymol blue) medium, as were three of the four isolates of the species *P. laurentii* (AM-0313, AM-0314 and AM-0315). The phenoloxidase test was negative for *C. bacillisporus* AM-316 and AM-317 after ten days of incubation on SSA plates. On the other hand, the *C. neoformans* AM-310 isolate started pigmentation on the tenth day of incubation; however, it did not have full pigmentation (Fig. 2). Those three isolated negatives for phenoloxidase were urea positive.

### MALDI-TOF identification

Sixty-four microorganisms were identified, from which 46 specimens of the genus *Cryptococcus* were isolated; 11 corresponded to other yeast species and seven to bacteria. Of the stool samples taken from the city of Tunja, 29 (315.18%) positive isolates belonging to the genus *Cryptococcus* were obtained, of which 23 (24.8%) corresponded to *C. neoformans,* 3 (3.22%) to *N.globosa*, 2 (2.2%) *N. albida*, 1 (1.07%) to *P. laurentii*. In addition to the findings, other yeasts such as *Candida albicans* (1), *Candida guillermondi* (5), *Candida parapsilosis* (1), *Candida tropicalis* (1) and *Rhodotorula mucilaginosa* (1), and some bacterial species such as *Bacillus subtillis* (4) and *Patoea agglomerans* (1) were identified. On the other hand, for the samples taken in the Ricaurte Alto region, a lower number of positive isolates was recorded, with a total of 17 (1.43%) positive samples for different species of the genus *Cryptococcus*, including *C. bacillisporus* (4), *C. neoformans* (2), *P. laurentii* (3), *N. albida* (2), *N. globosa* (5) and *P. flavescens* (1), in addition to finding yeasts such as *Rhodotorula mucilaginosa* (2) and two species of *Pseudomonas*; *Pseudomonas jesseni* (1) and *Pseudomonas oryzihabitans* (1).

### Molecular typification by RFLP of the URA5 gen

**Figure 3:**
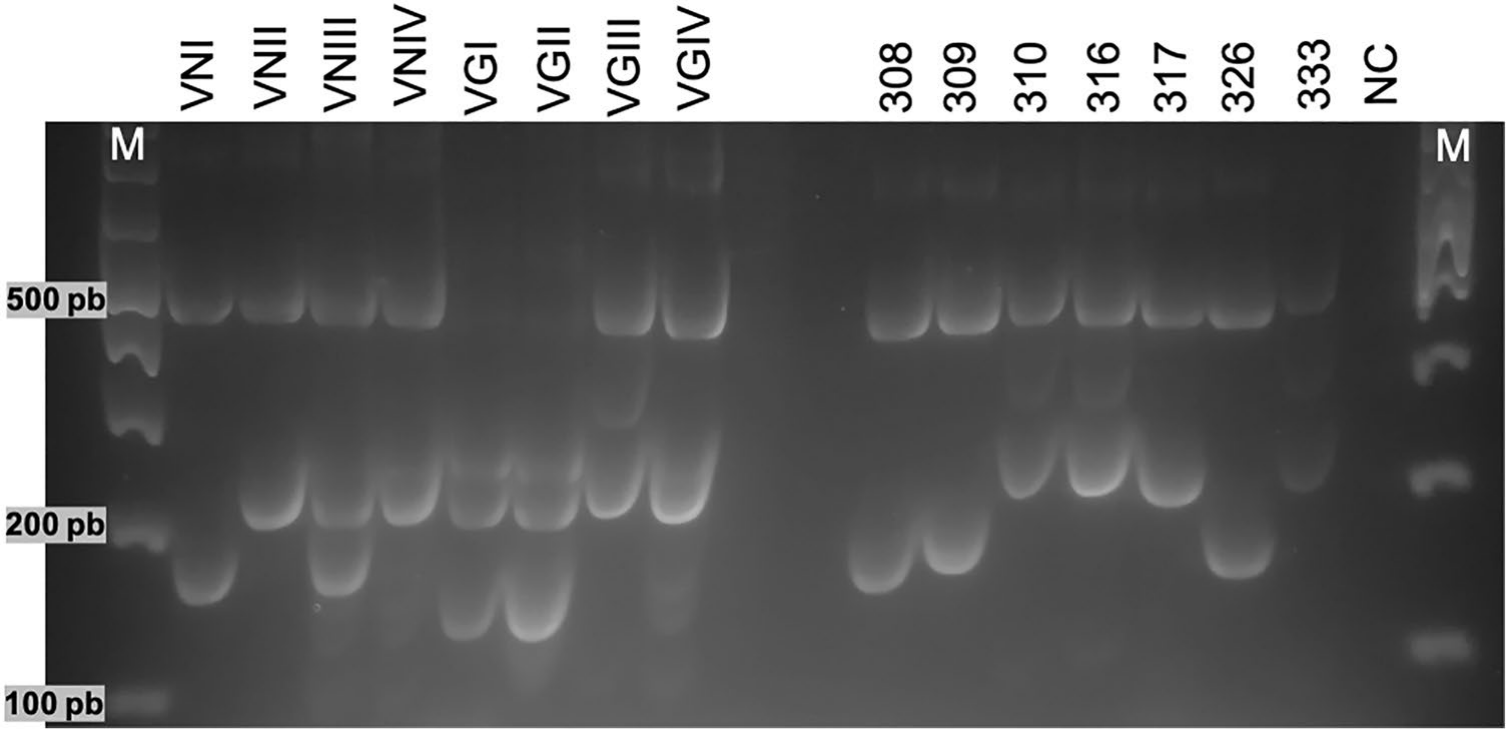
URA5-RFLP profiles obtained after digestion with the restriction enzymes HhaI and Sau96I in reference *Cryptococcus* spp*.* strains (lanes 2–9) and environmental isolates (samples AM-0308, AM-0309, AM-0310, AM-0316, AM-0317, AM-0326 and AM-0333). M, DNA size marker 1 kb Opti-DNA Marker Cat#G106. CN, negative control.

The molecular pattern was determined for the isolates identified as species of the *C. neoformans* and *C. gattii* species complex. Of the 25 isolates identified by MALDI-TOF MS as *C. neoformans*, 18 had a VNI molecular pattern, and 7 had a VNII molecular pattern. Likewise, for the four isolates identified as *C. gattii* species complex, three presented molecular pattern VGIII and one (AM-0317) molecular pattern VGIV contrary to typification by *URA5*-RFLP isolation AM-0317 was molecular pattern VGIII by sequencing of the ITS and LSU and MALDI-TOF (Fig. 3).

### Identification by sequencing ITS and LSU gen

Amplification of DNA samples with primers LROR and LR5 resulted in approximately 1,000 bp. The sequences were deposited in GenBank, and accession numbers were obtained (Table 1). Forty- six isolates belonging to the genus *Cryptococcus, Naganishia and Papiliotrema were identify*; *C. neoformans* (*n* = 25), *N. globosa* (*n* = 8), *N. albida* (*n* = 4), *C. bacillisporus* (*n* = 4) (*Cryptococcus bacillisporus* VGIII), *P. laurentii* (*n* = 4), and *P. flavescens* (*n* = 1). Also, nine species non-*Cryptococcus* genus, including *Meyerozyma guilliermondii* (*n* = 3) (Kurtzman and Suzuki 2010), (anamorph *Candida guilliermondii*, Langeron & Guerra 1938), *Meyerozyma* (*Pichia*) *guilliermondii* (*n* = 1), *Candida albicans* (*n* = 1), *Candida tropicalis* (*n* = 1), *Meyerozyma caribbica* (*n* = 1) and *Rhodotorula mucilaginosa* (*n* = 2).

**Table 1:** Collection number, data, locality, source, species, molecular type, and GenBank accession numbers of isolates yeasts.

Collection number UBCHM	Collected data (Month/Year)	Locality	Source	Taxon	Molecular type	Sequence length (LROR/LR5)	Gene Bank accession number LSU	Sequence length (ITS1/ITS4)	Gene Bank accession number ITS
AM-0272	Jan-18	Tunja	Pigeon feces	*Papiliotrema laurentii* (formerly *C. laurentii*)	N/A	769	OP076773	511	OP060634
AM-0275	feb-18	Tunja	Pigeon feces	*Cryptococcus neoformans*	VNI	859	OP076774	543	OP060635
AM-0276		Tunja	Pigeon feces	*Cryptococcus neoformans*	VNI	871	OP076775	541	OP060636
AM-0277	mar-18	Tunja	Pigeon feces	*Naganishia albida (*formerly *Cryptococcus albidus)*^*1*^	N/A	782	OP076776	588	OP060637
AM-0279		Tunja	Pigeon feces	*Cryptococcus neoformans*	VNII	807	OP076777	534	OP060638
AM-0282		Tunja	Pigeon feces	*Cryptococcus neoformans*	VNII	802	OP076778	530	OP060639
AM-0283		Tunja	Pigeon feces	*Cryptococcus neoformans*	VNII	766	OP076779	533	OP060640
AM-0284		Tunja	Pigeon feces	*Cryptococcus neoformans*	VNII	861	OP076780	544	OP060641
AM-0285		Tunja	Pigeon feces	*Cryptococcus neoformans*	VNII	865	OP076781	527	OP060642
AM-0286		Tunja	Pigeon feces	*Naganishia albida (*formerly *Cryptococcus albidus)*^*1*^	N/A	843	OP076782	593	OP060643
AM-0288	apr-18	Tunja	Pigeon feces	*Cryptococcus neoformans*	VNI	891	OP076783	547	OP060644
AM-0289		Tunja	Pigeon feces	*Cryptococcus neoformans*	VNI	806	OP076784	531	OP060645
AM-0290		Tunja	Pigeon feces	*Cryptococcus neoformans*	VNI	861	OP076785	527	OP060646
AM-0291		Tunja	Pigeon feces	*Cryptococcus neoformans*	VNI	848	OP076786	535	OP060647
AM-0292		Tunja	Pigeon feces	*Cryptococcus neoformans*	VNI	851	OP076787	532	OP060648
AM-0295		Tunja	Pigeon feces	*Cryptococcus neoformans*	VNI	896	OP076788	527	OP060649
AM-0297		Tunja	Pigeon feces	*Naganishia globosa (*formerly *C. saitoi)*	N/A	862	OP076789	610	OP060650
AM-0298		Tunja	Pigeon feces	*Naganishia globosa (*formerly *C. saitoi)*	N/A	827	OP076790	605	OP060651
AM-0299		Tunja	Pigeon feces	*Cryptococcus neoformans*^*1*^	VNI	771	OP076791	532	OP060652
AM-0300		Tunja	Pigeon feces	*Naganishia globosa (*formerly *C. saitoi)*	N/A	858	OP076792	607	OP060653
AM-0301	may-18	Tunja	Pigeon feces	*Cryptococcus neoformans* var* grubii*	VNI	859	OP076793	471	OP060654
AM-0302		Tunja	Pigeon feces	*Cryptococcus neoformans*	VNI	871	OP076794	542	OP060655
AM-0303	jun-18	Tunja	Pigeon feces	*Cryptococcus neoformans*	VNII	865	OP076795	542	OP060656
AM-0304		Tunja	Pigeon feces	*Cryptococcus neoformans*	VNI	870	OP076796	526	OP060657
AM-0305		Tunja	Pigeon feces	*Cryptococcus neoformans*	VNI	876	OP076797	525	OP060658
AM-0306		Tunja	Pigeon feces	*Cryptococcus neoformans*	VNI	800	OP076798	523	OP060659
AM-0307		Tunja	Pigeon feces	*Cryptococcus neoformans*	VNI	766	OP076799	523	OP060660
AM-0308	jul-18	Tunja	Pigeon feces	*Cryptococcus neoformans*	VNI	863	OP076800	526	OP060661
AM-0309		Tunja	Pigeon feces	*Cryptococcus neoformans*	VNII	867	OP076801	542	OP060662
AM-0310	jun-19	Sáchica	Hollowness	*Cryptococcus bacillisporus*	VGIII	874	OP076802	531	OP060663
AM-0312		Sáchica	Debris	*Naganishia albida* (formerly *Cryptococcus albidus*)^*1*^	N/A	799	OP076803	593	OP060664
AM-0313		Sáchica	Ground	*Papiliotrema laurentii* (formerly *Cryptococcus laurentii*)	N/A	885	OP076804	510	OP060665
AM-0314		Sáchica	Ground	*Papiliotrema laurentii* (formerly *Cryptococcus laurentii*)	N/A	868	OP076805	508	OP060666
AM-0315		Sáchica	Cortex	*Papiliotrema laurentii* (formerly *Cryptococcus laurentii*)	N/A	872	OP076806	507	OP060667
AM-0316		Sutamarchán	Leaves	*Cryptococcus bacillisporus*	VGIII	776	OP076807	526	OP060668
AM-0317		Sutamarchán	Leaves	*Cryptococcus bacillisporus*	VGIII/VGIV^2^	868	OP076808	495	OP060669
AM-0319	jul-19	Sáchica	Hollowness	*Papiliotrema flavescens* (formerly *Cryptococcus flavescens*)	N/A	801	OP076809	515	OP060670
AM-0322		Sáchica	Debris	*Naganishia globose* (formerly *Cryptococcus saitoi) *^*1*^	N/A	781	OP076810	613	OP060671
AM-0323		Sáchica	Cortex	*Naganishia albida* (formerly *Cryptococcus albidus*)^*1*^	N/A	871	OP076811	593	OP060672
AM-0325		Sutamarchán	Leaves	*Cryptococcus neoformans*^*1*^	VNI	800	OP076812	523	OP060674
AM-0326		Sutamarchán	Hollowness	*Cryptococcus neoformans*	VNI	804	OP076813	531	OP060673
AM-0328	Aug-19	Sáchica	Hollowness	*Naganishia globosa (*formerly *C. saitoi)*	N/A	866	OP076814	597	OP060675
AM-0329		Sáchica	Hollowness	*Naganishia globosa (*formerly *C. saitoi)*	N/A	802	OP076815	600	OP060676
AM-0330		Sáchica	Debris	*Naganishia globosa (*formerly *C. saitoi)*	N/A	874	OP076816	604	OP060677
AM-0331		Sáchica	Cortex	*Naganishia globosa (*formerly *C. saitoi)*	N/A	874	OP076817	604	OP060678
AM-0333	oct-19	Sutamarchán	Hollowness	*Cryptococcus bacillisporus*	VGIII	864	OP076818	526	OP060679

^1^The MALDI-TOF MS results were inconsistent with LSU and ITS sequencing results. ^2^The LSU and ITS sequencing and MALDI-TOF MS results (VGIII) were inconsistent with *URA5*-RFLP molecular type (VGIV). (N/A): not applicable.

**Figure 4:**
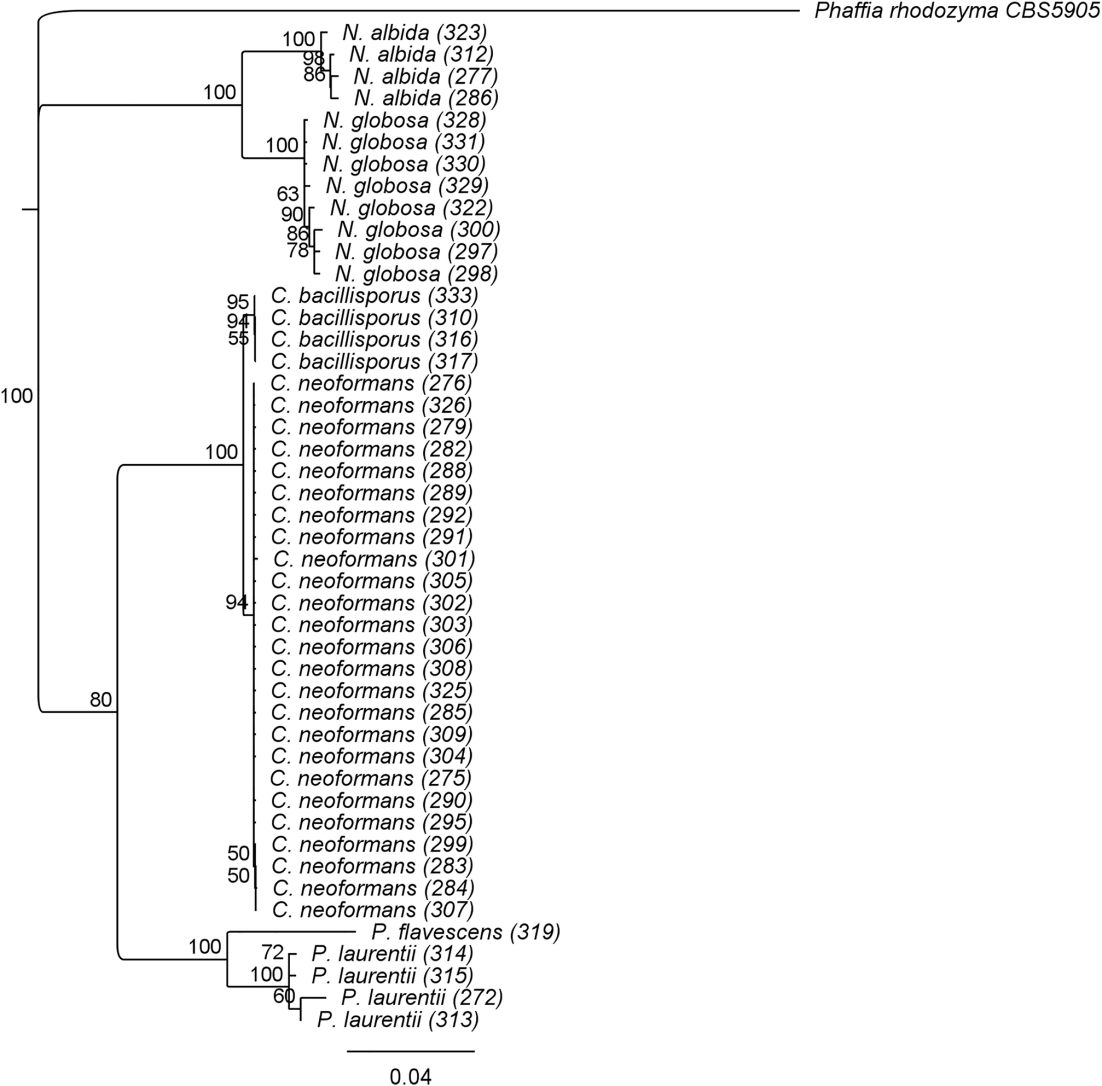
Phylogenetic tree of yeast strains identified in this study. Relationships were inferred using the neighbor-joining method in Geneious Prime® 2021.0.3 software. The analysis involved 46 nucleotide sequences and one outgroup with nucleotide sequences of *Phaffia rhodozyma* CBS5905. All positions containing gaps and missing data were eliminated. Numerical values above the internodes are the percentages of 1000 bootstrap replications. Bootstrap values greater than 50% are indicated. The scale bar of 0.04 represents nucleotide substitutions per position.

Concatenated ITS and LSU sequences with high bootstrap values generated by neighbor-joining analyses supported the differentiation of the sixclades: 1. *N. albida* (bootstrap values 100), 2*. N. globosa* (boot values 100), 3. *P. laurentii* (bootstrap values 100), 4. *P. flavescens* (boot values 100) 5. *C. bacillisporus* (bootstrap 95 values). 6. *C. neoformans* (boot values 94) (Fig. 4).

### Redundancy analysis (RDA) for stool samples of C. livia

**Figure 5:**
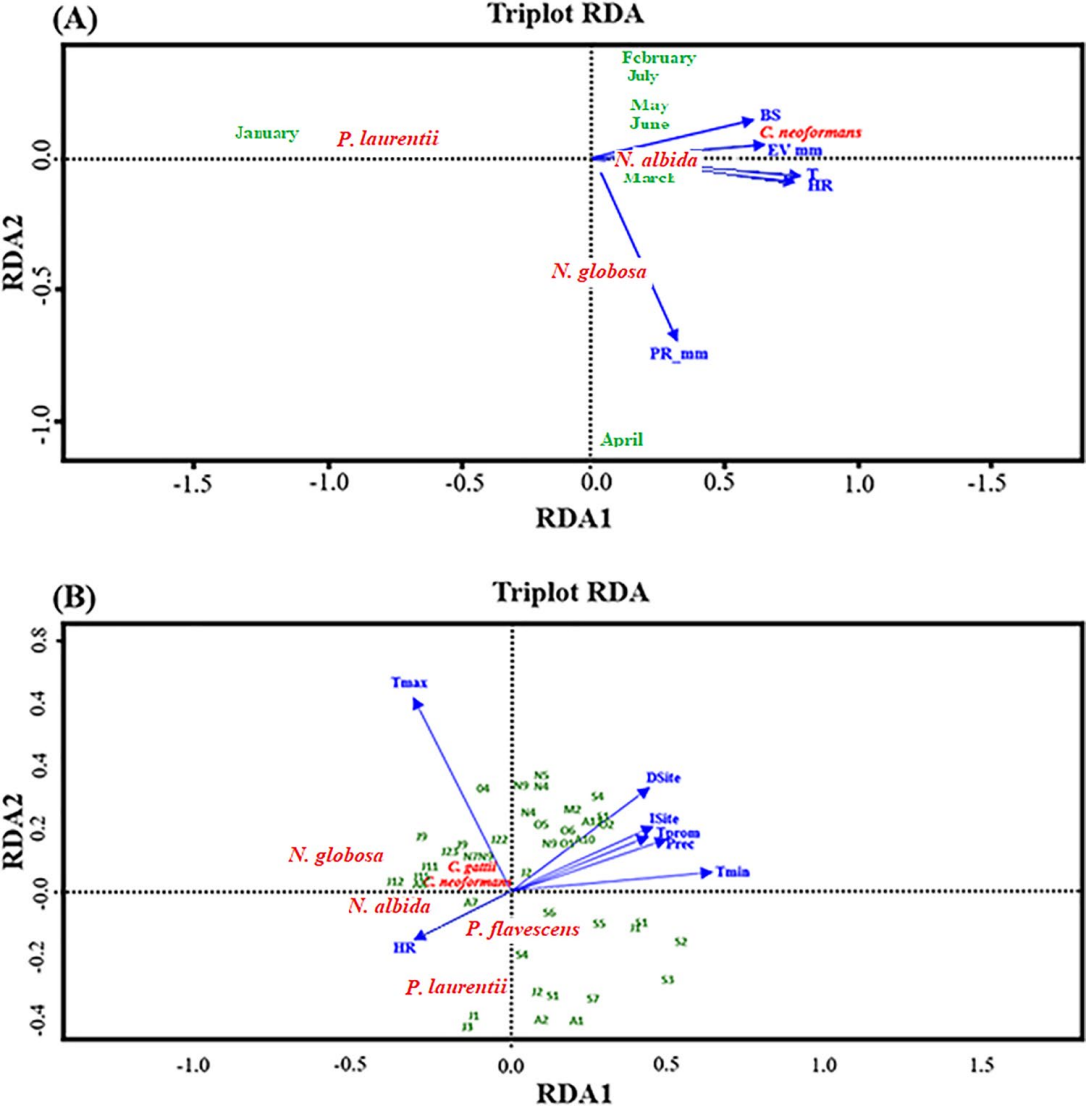
Redundancy analysis (RDA). (**a**) Triplot of the relationship between the presence of species of *Cryptococcus*, *Naganishia* and *Papiliotrema*. per month of sampling and the environmental parameters for the stool samples taken in the city of Tunja. (**b**) Triplot of the relationship between the presence of *Cryptococcus*, *Naganishia* and *Papiliotrema* species per sampling month and the environmental parameters taken from olive trees in the municipalities of Sáchica, Sutamarchán, and Villa de Leyva.

It is essential to mention that the data collected in Tunja were organized according to the sampling months, as can be seen in Fig. 5a for the stool samples. It is highlighted that the results indicate that the first three components explain the variability of 93.47% of the data.

The redundancy analysis established that the main variables that intensify the appearance of species were relative humidity, temperature, and precipitation. Relative humidity (RH) and temperature (T) positively correlated with the appearance of the species *N. albida* in March. The other environmental variables are positively related to the appearance of this species. Likewise, these environmental variables (HR, T, and P) negatively correlated with the *P. laurentii* species. As these variables increase, the probability of finding this species in the environment is lower.

For the *C. neoformans* species, the variables that have a positive correlation and favor its appearance in the environment are solar brightness (BS) with 76.46% and evaporation (EV_mm) with 80.3%, contrary to the negative relationship that was evidenced with the precipitation variable.

Finally, the species *N. globosa* presented a more significant relationship with the precipitation variable (PR_mm); it is more likely to find this species when precipitation is lower since its correlation with this analysis was 39%.

It should be noted that for the sampling carried out in the city of Tunja, direct and indirect light records were not made, only those mentioned above, which IDEAM provided.

### Redundancy analysis (RDA) for olive tree samples

It is essential to mention that to carry out this analysis, data was organized with codes where J was assigned to July, A to August, S to September, O to October, and N to November, as can be shown in Fig. 5b, for the data taken in Sutamarchán, Sáchica and Villa de Leyva.

The redundancy analysis allowed us to establish the main variables that intensify the appearance of *Cryptococcus, Naganishia* and *Papiliotrema* species; they were relative humidity and temperature.

The relative humidity (HR) is inversely related to the variables of indirect light (ISite), direct light (Dsite), average temperature (Tprom), and precipitation (Prec). According to the data collected, the relative humidity increases when ISite, Dsite, Tprom, and Prec variables decrease; then, the relative humidity decreases when the mentioned variables increase (Fig. 5b). The species *N. albida*, *P. laurenteii* and *P. flavescens* are found to a greater extent when the relative humidity of the medium is lower. In contrast, *C. neoformans and C. gattii* species complexes and *N. globosa* were isolated more frequently when the relative humidity was higher.

Likewise, the maximum temperature is also related to the appearance of species of the genus *Cryptococcus*, presenting a directly proportional relationship with the species of *C. neoformans* and *C. bacillisporus* and, to a lesser extent, *N. globosa*. These species were found between 20 and 25 °C, reported in July and August in the municipality of Sáchica. It should be noted that the variables of direct and indirect light, precipitation, minimum temperature, and average temperature were inversely related to the presence of all the species above.

### Multiple correlations analysis from samples obtained from stool samples

**Figure 6:**
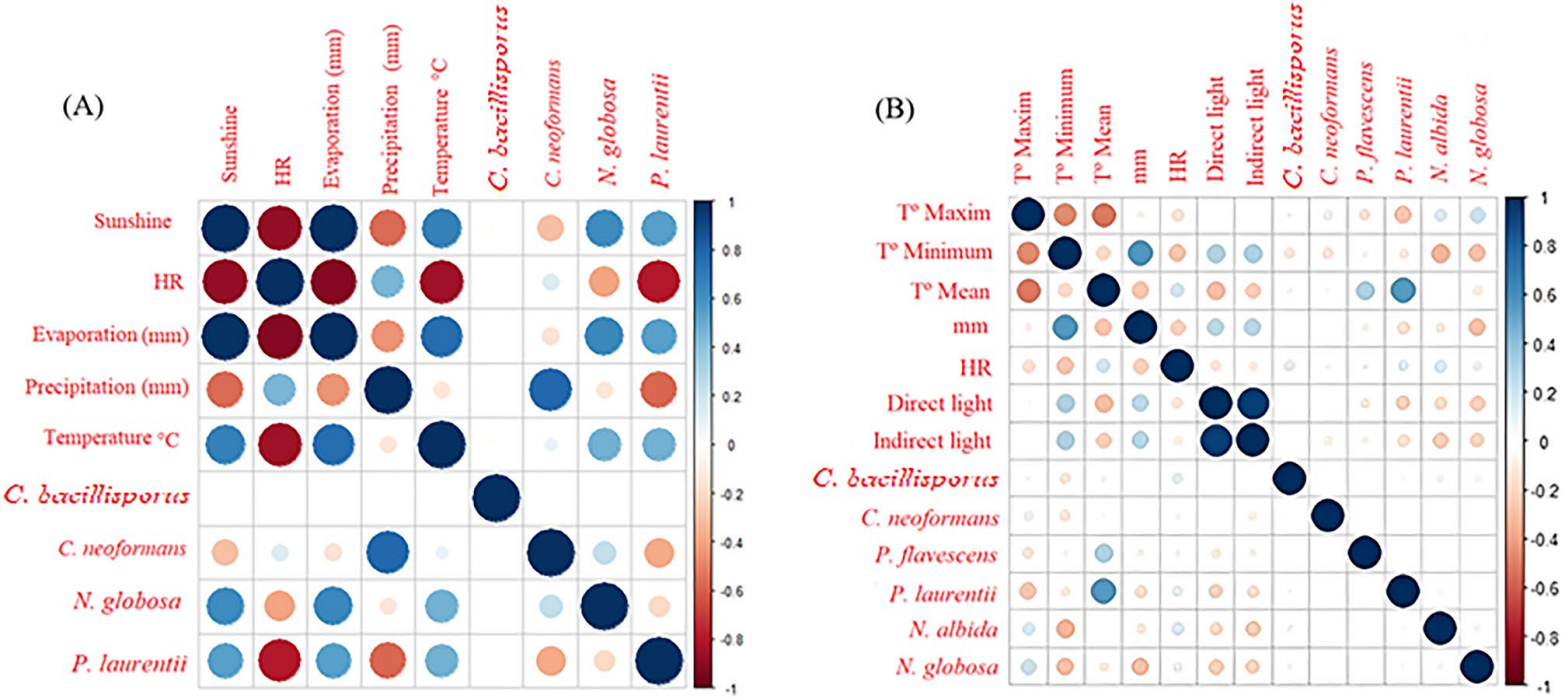
Multiple correlation analysis from samples obtained from pigeon feces (**a**) and samples from the Ricaurte Alto region (**b**). Color Intensity and cycle size describe the percentage of the relationship of the variables (right side of the figure). The colors on the graph represent positive correlation (Blue) and negative correlation (Red).

As shown in Fig. 6a, there is a positive correlation (78%) between the isolates of *C. neoformans* and precipitation; that is, precipitation contributed positively to obtaining isolates of this species; therefore, they are directly found related. Contrary to the negative correlation (30%) evidenced in the variable of solar brightness, that says, as the solar brightness increases, the probability of isolating *C. neoformans* in the environment decreases. Unlike what was found for *N. globosa,* which presents a positive correlation (62%), as the solar brightness increases, the probability of finding this species in the environment increases.

On the other hand, for *P. laurentii* a negative correlation of 80% with relative humidity was determined; that is, it is an inversely proportional relationship; therefore, as relative humidity increases, it is unlikely to find this species in the environment.

### Multiple correlations analysis from samples obtained from the Ricaurte Alto region

The variables of direct light (direct Site) and indirect (indirsite) present a positive correlation of 100%, with a relationship percentage of 1, which reflects that they are directly related. Similarly, C. *bacillisporus* has a slight positive correlation of 40% with relative humidity, which means that it is more likely to find this species when the relative humidity is higher. In the same way, a negative correlation of 30% can be observed between the appearance of *C. neoformans* and the minimum temperature; this is an inversely proportional relationship. As the temperature increased, it was more likely to isolate *C. neoformans* from the environment (Fig. 6b).

For the other species of *Cryptococcus*, it is essential to point out that *P. laurentii* has a positive correlation of 40% with the average temperature, thus indicating that this particular species is associated with average temperatures that range between 14 and 15 °C, which would indicate an association with cold to temperate places. In the case of the *N. albida* and *N. globosa* species, they present an inversely proportional correlation of 50% with the minimum temperature; according to our results, it is more likely to be found at temperatures above 15 °C.

When performing the logistic regression test, it was established that the specie that was most influenced by the environmental variables was *N. albida* with a p-value of 0.0078.

## Discussion

It is essential to point out that the objective of this research was to establish the first report of species of the *Cryptococcus* and *Cryptococcus*-like yeasts in the department of Boyacá. As well as to identify through microbiological and molecular tests and differentiate the species of *Cryptococcus* found. Similarly, establishing an association between environmental variables and the presence of species of *Cryptococcus, Naganishia and Papiliotrema* spp. In this way, the results permitted establishing the environmental distribution of this yeast in the Boyacá region, thus contributing to human health care.

Several studies have shown that *C. neoformans* has mainly pigeon feces as an environmental niche. This association is mainly due to the enzyme urease in *Cryptococcus* species that allows it to assimilate the nitrogen present in the medium. It should be noted that according to our results, *P. laurentii* isolates were negative for urease, which is also mentioned by Hoog De GS, et al. in (2000), and for the enzyme phenoloxidase in SSA medium, as reported by Pedroso et al.^69^. Likewise, as reported by Toplis et al.^70^, when the yeast grows at 37 °C and there is urea deficiency, low levels of melanin production are observed, which can be associated with low or no pigmentation in the SSA medium^70^. It should be noted that the phenoloxidase test was positive for the vast majority of *Cryptococcus spp.* isolates, except for strains AM-316, AM-317 and AM-310. The latter started pigmentation in the SSA medium on the tenth day of incubation; this may be due to mutations in the associated genes that prevent melanization^71, 72^. For the CGB test, all *C. bacillisporus* were positive, as were three isolates of *P. laurentii*, which showed a change in the CGB medium, as reported by Tay et al.^73^.

The isolates identified as *C. neoformans* complex were primarily obtained from the city of Tunja. The above, related to the presence of pigeons and the average temperature (14 °C), conditions that, according to Quintero, Rosario and Pfeiffer, contribute to generating an environment conducive to the survival of this species^18, 42, 74^. On the contrary, the species of the *C. gatii* complex was isolated from tree holes in olive trees, as reported by other authors^54, 59, 75–77^. However, the association of *C. gattii* species complex with olive trees constitutes the first report in Colombia. On the other hand, only 0.33% of the total samples collected (1188) corresponded to the species of *C. gattii*, which demonstrates the difficulty of isolating it from environmental sources, as published by Contreras et al.^78^ and Toro^75^, who reported very low percentages of *C. gattii* species complex isolates of 8% and 0.7%^75, 78^.

Of the species of the genus *Cryptococcus* different from the *C. neoformans* and *C. gattii* complexes, *P. laurentii* was isolated from pigeon feces and olive trees, showing a predilection for average temperatures typical of the Boyacá territory. *P. laurentii* has been reported from different tree species^79^ avian droppings and air samples^35, 80^. Similarly, this species has been isolated from clinical samples in patients with clinical conditions such as meningitis^15^, fungemia, and cryptococcosis, considering it a pathogenic species^81, 82^.

Meanwhile, *N. albida* has been reported as a pathogenic species for humans^15^ with a pathogenic behavior similar to *C. neoformans*^83^. In our study, this specie was isolated from tree debris and leaves from olive trees in the region of Ricaurte Alto and pigeon feces in Tunja city. In comparison, the environmental record had been reported from tree hollows and excreta in the city of Uberlandia, State of Minas Gerais, Brazil^62^.

*P. flavescens* was isolated from tree hollows in the region of Ricaurte alto, in the municipality of Sáchica and associated with temperate temperatures, the same as reported in the study published by Brito et al.^62^ that isolated this species from *Mangifera indica*. *P. flavescens* has also been isolated from the cerebrospinal fluid of a patient with AIDS^17^.

*N. globosa* was isolated from pigeon feces and olive trees, in a range of 9 °C–25 °C, contrary to reported by Butinar and collaborators^84^; who reported this specie from the Arctic soil (5 °C), specifically in the coastal glaciers Conwaybreen, Kongsvegen, and Austria Love ´nbreen and the interior glacier Austre Brøggerb-reen^84^. Likewise, Cornnell et al. (2008) and Singh et al.^64^ identified strains of *N. globosa* from glacial ice cores^63, 64^. These results allow recognizing the adaptation of *N. globosa* to different sources and its affinity to environmental variables such as low precipitation, average temperature, and low relative humidity, demonstrating new conditions that allow its development in our results.

Regarding the analysis of the relationship between the environmental variables and the species found from pigeon feces, it can be associated that the lower the brightness of the sun, the greater the probability of finding the species *C. neoformans*. The Tunja city registered solar brightness was low, varying between 180 h/month to 78 h/month, a characteristic associated with many *C. neoformans* isolates (*n* = 8). While in the region from Ricaurte Alto, only one sample of this species was isolated in the month with the lowest incidence of direct and indirect light, as reported by Ellis et al.^85^ and Rosario et al.^48^. Other variables that favor the presence of this species are; low temperatures and precipitation, together with an increase in humidity, as reported by other authors^18, 42, 85^. The same happens with *N. albida,* which presents the same characteristics with a greater predilection for average temperature and a positive relationship when relative humidity increases. While for *P. laurentii* it was established in this study that as all the variables decrease, the chance of finding it in the environment increases, contrary to what was reported by Pedroso et al.^80^, where, despite not specifying the variables studied, they relate it to a tropical climate^79, 80^.

For the isolates obtained from olive trees, as a first report, it was established that the species *N. albida*, *P. laurenteii*, and *P. flavescens* are isolated mainly in environments with low relative humidity. In this regard, the variable relative humidity was negatively correlated for the species mentioned above. On the other hand, a positive correlation was established for the maximum recorded temperature variable and *C. neoformans* and *C. gattii* species complex, equal to that reported for Bogotá city by Castañeda et al.^65^.

Furthermore, it is essential to note that the molecular patterns for the species of *C. neoformans* and *C. gattii* species complexes isolated in this study coincide with those reported for other departments of Colombia. The VNI molecular pattern is mainly reported in environmental isolations, followed by the VNII molecular pattern^86–89^. For the *C. gattii* complex the VGIII molecular pattern is reported more frequently, followed by VGII and VGI^77, 87, 88^. Additionally, the RFLP analysis identified the AM-0317 strain as VGIV but was classified as VGIII by MALDI-TOF and ITS/LSU sequences, the incorrectly grouping to VGIV can be the result of a point mutation in the RFLP restriction site. As reported by Trilles et al.^90^ and Firacative et al.^91^, a single nucleotide mutation resulting in the misidentification of isolates as VGIV, subsequently classified as molecular type VGIII by MLST analysis^90, 91^. In this respect, further phylogenetic analysis based on MLST and Whole Genome Sequencing (WGS) is required to establish the molecular structure of this strain.

Finally, molecular techniques using ITS and LSU allowed us to identify all isolates to the species level. However, it was observed that strains AM-0277, AM-0286, AM-0312, and AM-0323 identified as *N. albida* by LSU and ITS sequencing presented inconsistent results with MALDI-TOF which resulted in *N. liquefaciens*. Likewise, *C. neoformans* AM-0299 was identified as *N. globosa* by MALDI-TOF. Also, *N. globosa* (AM-0322) and *C. neoformans* AM-0325 were identified as *N. liquefaciens* by MALDI-TOF. Therefore, improving MALDI-TOF MS spectra libraries and implementing another characterization method to identify the species-level isolates is necessary.

## Conclusions

The findings of this study constitute the first report of *C. neoformans* and *C. gattii* species complexes, *P. laurentii, N. liquefaciens, N. globosa,* and *P. flavescens* in the department of Boyacá.

The data obtained from the microbiological identification were similar to those obtained by the molecular identification. The MALDI-TOF MS identification presented a correct recognition of the *C. neoformans* and *C. gattii* species complexes*,* including the molecular type. Most of the results obtained by *URA5*-RFLP were consistent with the other techniques employed, with only one atypical RFLP pattern identified as VGIV. Additionally, 89% of the non-neoformans/non-gattii *Cryptococcus* species, were correctly identify. In the same way, ribosomal subunit DNA sequencing allows to differentiate *C. neoformans* and *C. gattii*, however other *Cryptococcus* species requires more than one set of DNA markers.

In the Ricaurte Alto region, the environmental variables related to a higher recovery of *Cryptococcus; Naganishia and Papiliotrema* species were medium to high temperatures and the relative humidity of the environment.

For the sampling of *C. livia* feces carried out in Tunja, it is concluded that the environmental. conditions that favored the recovery of *C. neoformans* were low sunshine and increased precipitation.

For the species *P. laurentii* and *P. flavescens*, the first report of association with environmental conditions that favor their recovery, was made. In addition, the species *N. globosa* was reported to have a new favorable environment for its development.

## Methods

### Study area

**Figure 7:**
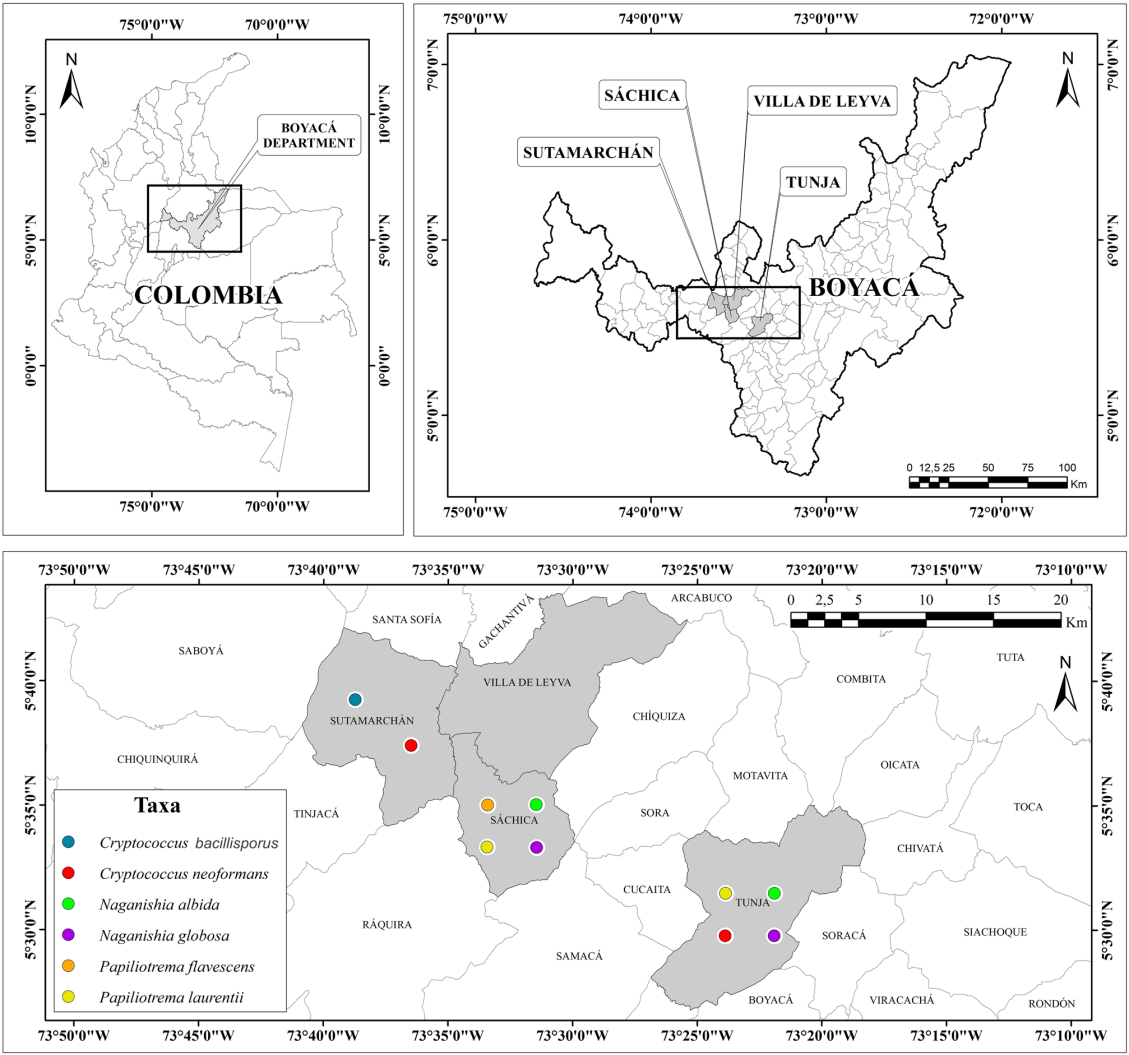
Map of environment isolation of *Cryptococcus, Naganishia and Papiliotrema* spp. from Boyacá- Colombia. MS: Map Scale. Source: Authors.

The first stage of the study was carried out in the city of Tunja, capital of the department of Boyacá—Colombia, for seven months, from January to July 2018. The sample collection was done in six points of Tunja city (República Forest, San Ricardo Forest, Escuela Normal Superior, Forest of the Universidad Pedagógica y Tecnológica de Colombia, Sugamux park and Plaza de Bolívar), which were selected for presenting an abundant avian population (Fig. 7). For the second stage, samples were collected for five months, from June to November 2019. The study area included the upper province of the department of Boyacá, made up of three municipalities, such as Villa de Leyva, located 40 km west of Tunja; Sáchica, located 32 km west of Tunja and Sutamarchán, which is 44 km from Tunja^92^. The selected sectors corresponded to areas with the presence of olive trees, such as; the central parks of Sáchica, Sutamarchán, the Nariño Park in Villa de Leyva, since they are a potential source of contamination and human influence, and the olive grove in the municipality of Sutamarchán, which is considered one of the oldest trees in the region (Fig. 7).

### Sample collection

A total of 93 samples of pigeon feces and 1211 from eucalyptus trees in Tunja and 1188 environmental samples from olive trees were collected. The sampling was random for each site, using biosecurity measures, to avoid the inhalation of viable spores. At the tree collection points, samples of bark, leaves, soil, cavities, and debris were taken, and stool samples were collected in different parts of the city. The samples were deposited in hermetically sealed bags, transferred and processed in the Biological Sciences Laboratory of the University of Boyacá.

*Eucalyptus globulus* and *Olea europea,* the species associated with the samples obtained in this study, are not endangered. The sample collection is non-lethal for the plants because it includes non-living parts of the plant such as bark, fallen leaves, soil, cavities, and debris^36^.

The sample collection complied with relevant institutional, national, and international guidelines and legislation. The permission to collect biological samples was granted by Autoridad Nacional de Licencias Ambientales—ANLA (Resolution No 01300 de 2019).

### Environmental data

In the sampling points associated with trees, environmental data were collected through what was reported by the Institute of Hydrology, Meteorology, and Environmental Studies of Colombia. Canopy cover was evaluated using the WinCanopy equipment (canopy structure and solar radiation, with cover and light variables), which allowed characterizing the habitat where *Cryptococcus* species isolated from trees are found.

### Microbiological identification

During a maximum of 24 h after collecting the samples, processing of the samples was carried out following the protocol described by Escandon et al. (2010), using the extraction technique with phosphate-buffered saline (PBS)^77^. Briefly, 1 g of each pigeon dropping sample and 5 g of soil, bark, leaves, cavities, and debris were suspended in 50 ml of PBS 1X with 0.2 g of chloramphenicol, followed by manual shaking for 5 min. After resting 30 min, 100 µl of the supernatant was sown in sunflower seed agar (SSA) medium plates (Khan et al. 2004). The plates were then incubated at 25 °C and checked daily for brown colonies for five days. Each phenol oxidase-positive/brown colony was sub-cultured on Sabouraud agar for purification and phenotypical characterization.

All single isolated colonies, not only pigmented colonies, were tested for the presence of capsules by India ink examination, urease production on urea agar^93^ and thermotolerance at 37 °C on Sabouraud agar. The species *C*. *neoformans* and *C*. *gattii* were differentiated on canavanine-glycine-bromothymol blue (CGB) medium^94, 95^. Additionally, the colonies that did not pigment in SSA agar despite presenting a capsule, positive urea, and growth at 37 °C, were seeded again in SSA agar and incubated for ten days with daily observation. *C. albicans* ATCC 10,231 and *C. neoformans* ATCC 32,045 were used as negative and positive controls of the phenol oxidase test.

### Matrix-assisted laser desorption/ionization time-of-flight (MALDI-TOF)

To obtain protein profiles for each yeast isolate, these were seeded in Sabouraud agar and incubated for 24 h at 37 °C. An inoculum of each pure culture was deposited in a well of a metal plate for analysis (Bruker Daltonics®), formic acid was added twice, allowing it to dry between each application. Then, 1μL of the matrix was added (2.5 mg/mL of α-cyano-4-hydroxycinnamic acid HCCA Bruker Daltonics®) in 50% acetonitrile, 2.5% trifluoroacetic acid, and 47.5% HPLC Water-Sigma). The mixture was allowed to dry at room temperature. The spectra for each isolate were obtained after 240 laser shots in six different regions within the well by a Microflex spectrometer LT MALDI-TOF MS (Bruker Daltonics) and analyzed using Bruker Flex software and MALDI Biotyper RTC 3.0 (Bruker Daltonics®). The BTS standard (Bacterial Test Standard) *Escherichia coli* DH5 from alpha peptide was used as a calibration standard. All the spectra were analyzed from 2,000 to 20,000 Da and then compared with the BDAL database provided by Bruker. All protein profiles were used to make a correlation dendrogram using RTC Biotyper 3.0 software^96^.

An extraction process was carried out for the isolates that did not present protein profiles, which is briefly described below; 300 μL of HPLC water was added to a 1, 5 ml tube, after which several isolated colonies were transferred to the tube, and vortexed, 300 μL of ethanol was added and turned to pass by vortex, it was centrifuged for 2 min at 1400 rpm, the ethanol was decanted, and it was centrifuged again with the same conditions. The excess ethanol was stirred with the pipette, and 10 μL to 50 μL of formic acid was added, depending on the formed pellet, and vortexed, 10 μL to 50 μL of acetonitrile was added, it is essential to note that the amounts of formic acid and acetonitrile must be in identical volumes, it was centrifuged again for 2 min at 1400 rpm. 0.85 μL of the supernatant was pipetted into a well of the MALDI-TOF plate. Avoid touching the pellet at the bottom and allow it to dry. The sample was covered with 0.85 μL of the matrix and dry.

## Molecular identification

### DNA extraction

The Wizard® Genomic DNA Purification Kit protocol from Promega was used with some modifications. The isolates belonging to the genus *Cryptococcus* were plated 48 h in advance on yeast extract, peptone, dextrose (YEPD) agar, at a temperature of 37 °C, then 0.9 to 0.12 g of the culture was transferred to a 1.5 ml Eppendorf tube containing 293 µL of EDTA (50 mM). Subsequently, 0.10 g of glass beads were added and subjected to vortex agitation for 10 min, the supernatant was transferred to a clean tube, and the procedure indicated by the extraction kit, described for yeasts. The quantification of DNA was made with QuantiFluor® dsDNA System (Promega).

### Polymorphisms of the URA5 gene by RLFP

Molecular type was determined by RFLP analysis of the *URA*_*5*_ gene. This *URA*_*5*_ gene was amplified with the two primers URA5 (5'ATGTCCTCCCAAGCCCTCG ACTCCG 3') and SJ01 (5'TTAAGACCTCTGAACAC-CGTACTC 3'). PCR amplification of the *URA*_*5*_ gene was performed as described by Meyer et al.^38^, which was carried out with one cycle of 94 °C for the initial denaturation of 2 min, for 35 cycles in a thermocycler brand (AXYGEN MAXGYGENE) as follows: 94 °C for denaturation for 45 s, 1 min annealing at 61 °C and 2 min extension at 72 °C, followed by a final extension cycle for 10 min at 72 °C. The amplification products were visualized on 1.5% agarose gels in 1X TBE buffer stained with SafeViem™ Classic Cat. G108 0.3 mg/ml, at 100 V for 1 h. Subsequently, 30 μl of each PCR product was digested twice with Sau96I (10 U/μL) and HhaI (20 U/μL) for three hours and separated by 3% agarose gel electrophoresis at 90 V for five hours. The RFLP patterns were visually assigned by comparing them with the patterns obtained from the standard strains (VNI-VNIV and VGI-VGIV) provided by the Colombian National Institute of Health^38, 97^.

### PCR amplification and Sequencing of rLSU region

Two nuclear loci were amplified, the long subunit of ribosomal RNA (*LROR*: 5' -ACCCGCTGAACTTAAGC-3' *LR5:* 5' -ATC CTG AGG GAA ACT TC-3') and nuclear ribosomal internal transcribed spacer region (ITS1: 5' -CTTGGTCATTTAGAGGAAGTAA-3' ITS4: 5' -GGAAGTAAAAGTCGTAACAAGG-3'). Amplification followed the procedure reported by Gardes and Bruns^98^ and Vilgalys and Sun^99^ with some modifications. The reactions were carried out in 30 µL. The PCR mix contained primers at a final concentration of 0.5 µM, 1 µL of genomic DNA (2 ng/µL), and 12.5 µL of 2X PCR MasterMix (Applied Biological Materials Inc. (Abm)). The amplification program consisted of one initial cycle of 3 min at 94 °C, followed by 35 cycles comprising denaturation (1 min at 94 °C), annealing (30 s at 56 °C), and extension (1 min at 72 °C), and then a final extension (7 min at 72 °C). The amplified products were analyzed on 1% agarose gels stained with SYBR® Green (Applied Biological Materials Inc. (Abm)).

The PCR products obtained were purified and sequenced using the Sanger platform. The sequences obtained were edited using Geneious Prime® 2021.0.3 software; subsequently, with the consensus sequences, BLAST was performed in the GenBank database and MycoBank databases to determine the species or genus of each isolate. The identification presented in this article corresponds to the better similarity and overlap percent. The assembled sequences were submitted to the GenBank Database.

The isolates were preserved in glycerol 10% at − 80 °C and deposited in the Culture Collection of Fungi and Microorganisms of the University of Boyacá.

### Statistical analysis

Statistical analyzes were performed with the R studio version 4.1.1 program to find the relationship between environmental variables and the incidence of the fungus in the region, firstly, with descriptive statistics for PCA and RDA and basic statistics and probabilities to perform correlations and multivalent statistics, in this case, the logistic regression (to determine the variable of more weight) for the samples taken from olive trees. Multiple correlation was done to with the use of a matrix of 64 data and eight variables, among which are positive isolates (postcryto) for *Cryptococcus*, maximum temperature (Temmax), minimum (Temmin) and average (Tempro), relative humidity (RH), precipitation, direct light (Directside), and indirect (indirectside)^100^. Rstudio software, with packages (FactoMineR, factoextra, readxl, performanceAnalitytics, ggplot2, MVN) was done for this analysis.

Logistic regression was used to predict the probability of finding *Cryptococcus spp*. positive in association with the environmental variables studied, which are maximum (Temmax), minimum (Temmin) and average (Tempro) temperature, relative humidity (RH), precipitation, direct light (Directside), and indirect (indirecside), for this the analysis was carried out using the Rstudio program, with the packages (corrplot, ggplot2) (Chitarroni 2002)^101^.

RDA redundancy analysis was done to evaluate the presence of *Cryptococcus* species during the sampling months, taking into account the aforementioned environmental variables.

### Supplementary Information

Supplementary Legends.

Supplementary Figure 1.

Supplementary Figure 2.

Supplementary Tables.

## Data Availability

All data generated or analysed during this study are included in this published article. The sequence data have been deposited in GeneBank with the accession codes list in a Table 1.

## Abbreviations

AIDS: Acquired immunodeficiency syndrome
ATCC: American type collection culture
BS: Solar brightness
CGB: Canavanine-glycine-bromthymol blue agar
EDTA: Ethylenediaminetetraacetic acid
IDEAM: Instituto de Hidrología, Meteorología y Estudios Ambientales
ITS: Internal transcribed spacer
LSU: Large subunit
MALDI-TOF: Matrix-assisted laser desorption/ionization time-of-flight
MLST: Multilocus sequence typing
PBS: Phosphate-buffered saline
PPFD: Photosynthetic photon flux density
RDA: Redundancy analysis
RFLP: Restriction fragment length polymorphism
RH: Relative humidity
SSA: Sunflower seed agar
UBCHM: Universidad de Boyacá Colección de Hongos y Microorganismos
WGS: Whole genome sequencing
YEPD: Yeast extract, peptone, dextrose agar
