# Supplementary material for: First report of environmental isolation of Cryptococcus and Cryptococcus-like yeasts from Boyacá, Colombia
Source: Sci Rep. 2023 Sep 21;13:15755. doi: 10.1038/s41598-023-41994-6 (PMC10514045; doi:10.1038/s41598-023-41994-6)
Supplement: Supplementary file 1 — Supplementary Legends. [file 41598_2023_41994_MOESM1_ESM.docx]

**Supplementary figures legends**

**Supplementary Figure 1**. URA5-RFLP profiles obtained after digestion with the restriction enzymes HhaI and Sau96I in reference *Cryptococcus* spp. strains (lanes 2–9) and environmental isolates (lines 10-19) (samples AM-0275, AM-0276, AM-0282, AM-0279, AM-0283, AM-0284, AM-0285, AM-0288, AM-0289 and AM-0290). M, DNA size marker 1kb Opti-DNA Marker Cat#G106.

**Supplementary Figure 2.** URA5-RFLP profiles obtained after digestion with the restriction enzymes HhaI and Sau96I in reference *Cryptococcus* spp. strains (lanes 2–9) and environmental isolates (lines 10-19) (samples AM-0291, AM-0292, AM-0295, AM-0301, AM-0302, AM-0303, AM-0304, AM-0305, AM-0306 and AM-0307). M, DNA size marker 1kb Opti-DNA Marker Cat#G106.
