# Supplementary figures and images for: First report of environmental isolation of Cryptococcus and Cryptococcus-like yeasts from Boyacá, Colombia
Source: Sci Rep. 2023 Sep 21;13:15755. doi: 10.1038/s41598-023-41994-6 (PMC10514045; doi:10.1038/s41598-023-41994-6)

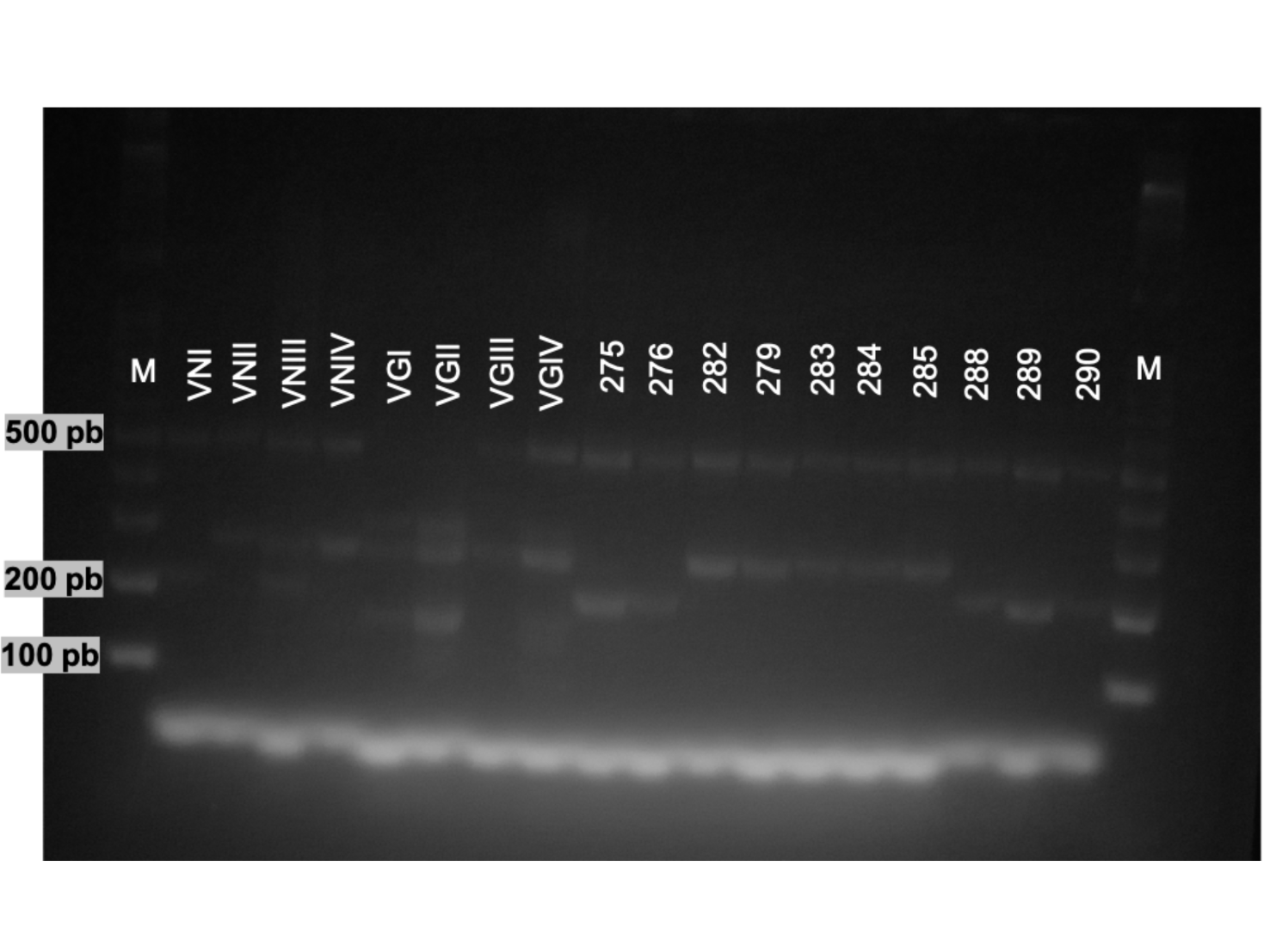

Supplement: Supplementary file 2 — Supplementary Figure 1. [file 41598_2023_41994_MOESM2_ESM.png]

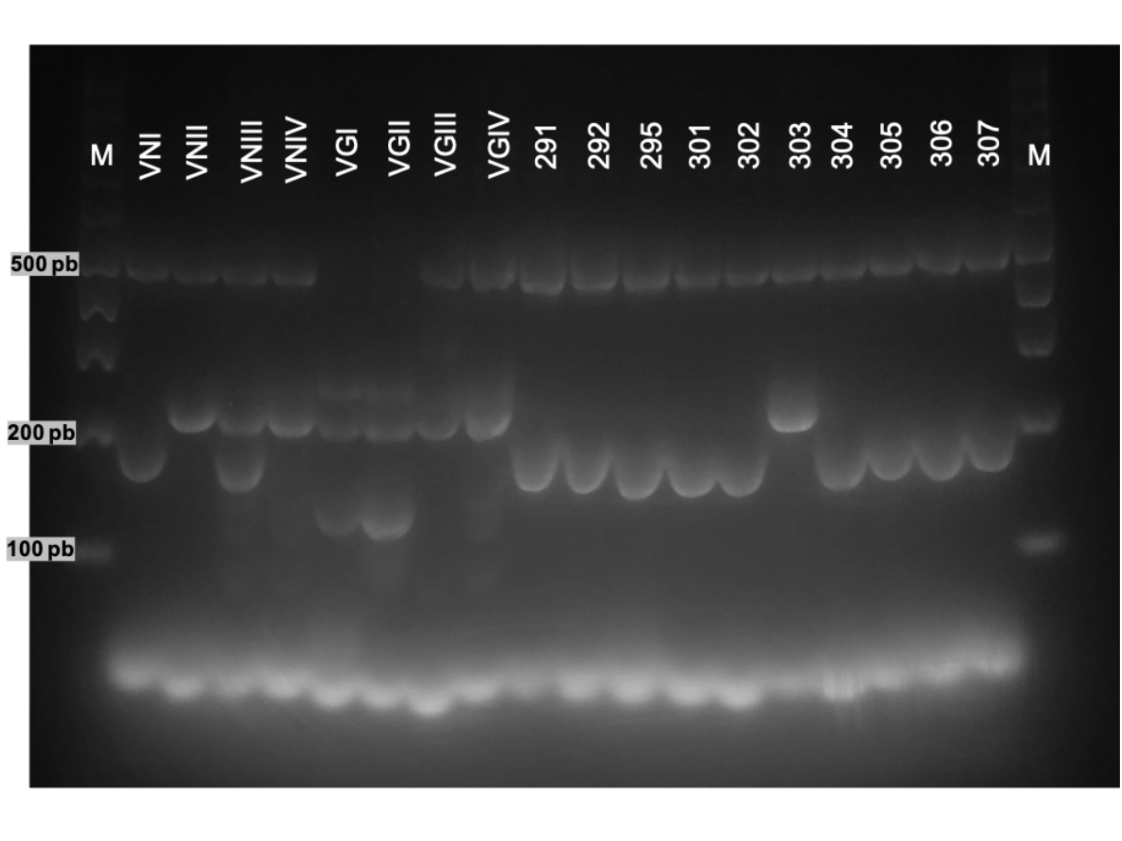

Supplement: Supplementary file 3 — Supplementary Figure 2. [file 41598_2023_41994_MOESM3_ESM.png]
